# Integrated system architecture with mixed-reality user interface for virtual-physical hybrid swarm simulations

**DOI:** 10.1038/s41598-023-40623-6

**Published:** 2023-09-07

**Authors:** Chuanqi Zheng, Annalisa Jarecki, Kiju Lee

**Affiliations:** 1 Mechanical Engineering, Texas A&M University, College Station, 77845 TX USA; 2 Engineering Technology & Industrial Distribution, Texas A&M University, College Station, 77845 TX USA

**Keywords:** Engineering, Mathematics and computing

## Abstract

This paper introduces a hybrid robotic swarm system architecture that combines virtual and physical components and enables human–swarm interaction through mixed reality (MR) devices. The system comprises three main modules: (1) the virtual module, which simulates robotic agents, (2) the physical module, consisting of real robotic agents, and (3) the user interface (UI) module. To facilitate communication between the modules, the UI module connects with the virtual module using Photon Network and with the physical module through the Robot Operating System (ROS) bridge. Additionally, the virtual and physical modules communicate via the ROS bridge. The virtual and physical agents form a hybrid swarm by integrating these three modules. The human–swarm interface based on MR technology enables one or multiple human users to interact with the swarm in various ways. Users can create and assign tasks, monitor real-time swarm status and activities, or control and interact with specific robotic agents. To validate the system-level integration and embedded swarm functions, two experimental demonstrations were conducted: (a) two users playing planner and observer roles, assigning five tasks for the swarm to allocate the tasks autonomously and execute them, and (b) a single user interacting with the hybrid swarm consisting of two physical agents and 170 virtual agents by creating and assigning a task list and then controlling one of the physical robots to complete a target identification mission.

## Introduction

A robotic swarm comprises many relatively simple and inexpensive robots collaborating and operating as a cohesive system. Although individual robots have limited sensing, communication, and processing capabilities, the collective swarm is expected to execute complex tasks such as pattern formation, object clustering, environment exploration, and target localization^1^. This unique capability makes it suitable for a wide range of applications, including agriculture, industry, space exploration, and military operations^2^. These applications benefit from the scalability, redundancy in information collection, and robustness to handle high levels of uncertainty provided by robotic swarms.

Despite its potential, swarm robotics research has predominantly been limited to low-fidelity simulations and constrained laboratory experiments. Simulations often employ simplified models of robots and environments, lacking in-depth physics and dynamics, which may lead to results that do not accurately predict real-world performance^3, 4^. Physical experiments are typically conducted in controlled laboratory settings using a small number of robots with limited mobility and functionality or with a large number of microrobots restricted to tabletop movements^5–7^. These setups fail to capture the complexities of real-world applications.

The development and operation of a robotic swarm pose distinctive challenges due to the increased complexity of the hardware and software involved. These challenges are further amplified by the large number of individual robots and the necessity of seamless interaction among them. Moreover, creating an effective interface between the swarm and the user(s) demands unconventional approaches compared to traditional human-robot interfaces. Depending on the desired level of autonomy and the nature of tasks, the user’s role can range from task planning to direct control over the entire swarm, a subset of the swarm, or even individual robots in situations requiring real-time collaborative efforts.

This paper introduces a system architecture that combines a Hybrid Robotic Swarm (HyRoS) with MR-based user interface (UI). The architecture facilitates the development, implementation, and evaluation of swarm robotic systems and associated technologies. The hybrid swarm comprises physical and virtual agents that coexist in the physical environment and possess communication and information-sharing capabilities. The proposed architecture is designed to be flexible and scalable, accommodating various swarm agent compositions and quantities. It also enables different levels of human–swarm interaction (HSI), ranging from swarm task planning to real-time control of individual robots for single or multiple users.

The remaining sections of the paper are structured as follows. In Section “System overview”, an overview of the system and the relevant technologies is provided. Section “Embedded swarm algorithms” delves into the swarm algorithms integrated into the HyRoS system. The design of the HSI is explained in Section “Hybrid swarm system and user interface”, and finally, Section “System demonstrations” showcases the experimental demonstrations. Prior to that, we discuss the existing literature on topics such as robotic swarm simulation, hybrid physical-virtual systems, and MR-based UI.

### Swarm simulators and hybrid physical-virtual swarms

Simulation plays a crucial role in robotics, serving various purposes such as analyzing kinematics and dynamics, developing control algorithms, offline programming, and designing mechanical structures^8^. Simulation involves creating a virtual representation that mimics the operation of the robots under different internal and external conditions. The ultimate goal is to predict the real-world performance of the robots or their components. However, the hardware, software structures, and control strategies of a robotic swarm differ significantly from those of a single-robot system. The complexity of developing, controlling, operating, and utilizing a swarm system increases exponentially due to the large number of robots involved^9, 10^. While single-robot systems are often designed for specific applications with relatively well-defined tasks, robotic swarms are expected to handle significant uncertainties in both tasks and operating environments^10^. Consequently, simulating a robotic swarm has typically focused on limited aspects compared to other areas of robotics.

Some existing swarm simulation tools include SwarmLab, ARGoS, Swarm-Sim, SwarmLab, and MATLAB with Simulink. SwarmLab is a MATLAB-based swarm simulation software, utilized to test decentralized swarm algorithms and assess their impact on scalable swarm movement^11–13^. ARGoS, another simulation platform, was used to examine swarm consensus time and probability for selecting environmental identification options^14, 15^. Swarm-Sim allows for 2D or 3D simulations of swarms to observe interactions among the agents in virtual environments^16–18^. MATLAB and Simulink were also employed to evaluate convergence and stability in an observer-based trajectory synchronization method, optimizing gains for each observer component^19^. These software tools provide researchers with valuable means to explore swarm dynamics and behaviors in a controlled and reproducible environment.

Although simulations have proven valuable in predicting various aspects of swarm capabilities, the disparity between simulations and physical implementations is more pronounced in swarm systems than in single-robot systems. Due to computational limitations and complexity, physical properties and dynamic characteristics are often simplified or even disregarded in simulations^4, 20^. Additionally, robot-to-robot (R2R) communication is often modeled as a binary variable, assuming either perfect or no communication at all^4^. As a result, many swarm control algorithms validated in simulations either fail in real-world scenarios or have not been implemented in physical swarm systems at all^21^. This highlights the need for bridging the gap between simulation and physical implementation to ensure the robustness and reliability of swarm behaviors.

A hybrid virtual-physical swarm, as described in this paper, refers to a system that combines physical swarm platforms with simulated capabilities or involves a combination of physical and virtual agents. This approach provides researchers with opportunities to bridge the gap between simulations and reality. For instance, in one study, a group of small e-puck robots was placed in an arena with a ceiling-mounted webcam for localization, and virtual sensor data were sent to each robot to simulate their responses to the environment^22^. Another work allowed users to control either the physical or virtual environment surrounding the swarm to observe the agents’ reactions^23^. In these cases, the robotic agents were physical, but the environment was simulated, enabling testing of physical hardware in various simulated environments and stimuli that are challenging to reproduce in the real world. Hybrid virtual-physical swarms can also be utilized to evaluate swarm algorithms with an increased number of members. For example, a combination of physical and virtual quadcopters was employed to simulate an entire swarm, where the physical quadcopters interacted with the virtual ones and responded to simulated stimuli^24^. This approach allows for testing the swarm algorithm’s response and behavior when scaling up the number of members.

### Mixed-reality based UI for swarm simulators

In contrast to the interaction expected between a user and a single robot, HSI often encompasses multi-level and interchangeable interactions. The user may need to engage with the entire swarm, a subset of the swarm, or even an individual agent within a specific task. Traditional UI modalities, such as 2D display visualization with keyboard and mouse input, may not provide the necessary flexibility and adaptability for such operations. To address these challenges, recent studies have explored innovative approaches to enable efficient and intuitive UI for swarm systems. These approaches aim to enhance the usability and interaction experience between the user and the swarm, allowing for more effective and seamless control and coordination.

A previous work proposed direct manipulation of physical objects for swarm formation and motion control in 2D scenarios^25^. Another study utilized a 3D haptic device to directly control swarm agents for obstacle avoidance^26^. MR has also been explored as a UI modality. In one demonstration, MR was used to control a robot arm and a mobile platform, synchronizing their coordinate systems using Microsoft’s HoloLens and the mobile robot’s LiDAR sensor^27^. In another study, an integrated system of UGV, UAVs, and a humanoid-type robot was controlled via HoloLens, with visual feedback from a multi-camera system for motion control^28^. However, this study lacked GPS input, limiting its use in uncontrolled environments or remote locations, and the system was not geographically synced with the real world without GPS data.

HoloLens’s spatial mapping capabilities enable the alignment of virtual and physical worlds. A previous study visualized the paths of UAVs in MR using HoloLens and interfacing with ROS for trajectory debugging^29^. However, the use of extra trackers limited its replication outside of controlled environments. Another system, ARENA, proposed using augmented reality for warehouse swarm operations with WsBots, visualizing the environment and swarm agents’ positions^30^, but it is still in the planning stage. The α-SWAT interface is an adaptable human–swarm teaming system integrating a virtual swarm simulator with multiple UI modalities for different users, including those with limb loss^31^. In this work, the UI modalities included MR display (Hololens), gesture inputs (camera or electromyography), and tactile feedback (vibration motor or implanted peripheral nerve interface). While the system supported user interaction and task execution with a virtual swarm overlaid in the real environment, it did not facilitate simultaneous operation and control of virtual and physical robots.

MR-based UIs demand precise hologram alignment within physical environments to ensure an optimal experience. Real-time interaction requires minimal communication latency when displaying dynamically changing holograms through MR devices. Several other technical challenges exist, such as limited field of view, localization/tracking restrictions, especially during user movement, which have been discussed in previous works^29, 32–34^. A recent study scrutinizing the accuracy of HoloLens 2’s inertial measurement unit (IMU) revealed that the largest measured error was merely 0.87% of the device’s total localized motion without camera tracking mechanisms^35^. Additionally, tests on the hand-tracking capabilities of HoloLens 2 demonstrated an impressive accuracy within the range of 1.5–3.0 cm^36^. In terms of overlaying virtual holograms onto physical objects in the environment, HoloLens 1 exhibited an average alignment error of 12.4 mm in surgical settings^37^, while HoloLens 2 showed significant improvement with a maximum root-mean-square error (RMSE) of 3.018 mm in orthopedic cases, mainly attributed to enhanced depth perception capabilities^38^.

The presented HyRoS system occupies a unique intersection of virtual-physical hybrid swarms and MR-based UI design. It addresses some of the main challenges of using MR and swarm control interfaces. The system enables an accurate graphical representation of the simulated swarm members by syncing the virtual environment with the real physical geography. It offers a fully integrated hybrid swarm system that allows users to plan, interact, and control both virtual and physical robotic agents. The system supports the same data processing and visualization for both types of agents through an MR display. HoloLens 2 was adopted as the MR-based UI modality considering its demonstrated utilities. The UI supports different user roles, such as planner and observer, similar to^31^.

## System overview

**Figure 1:**
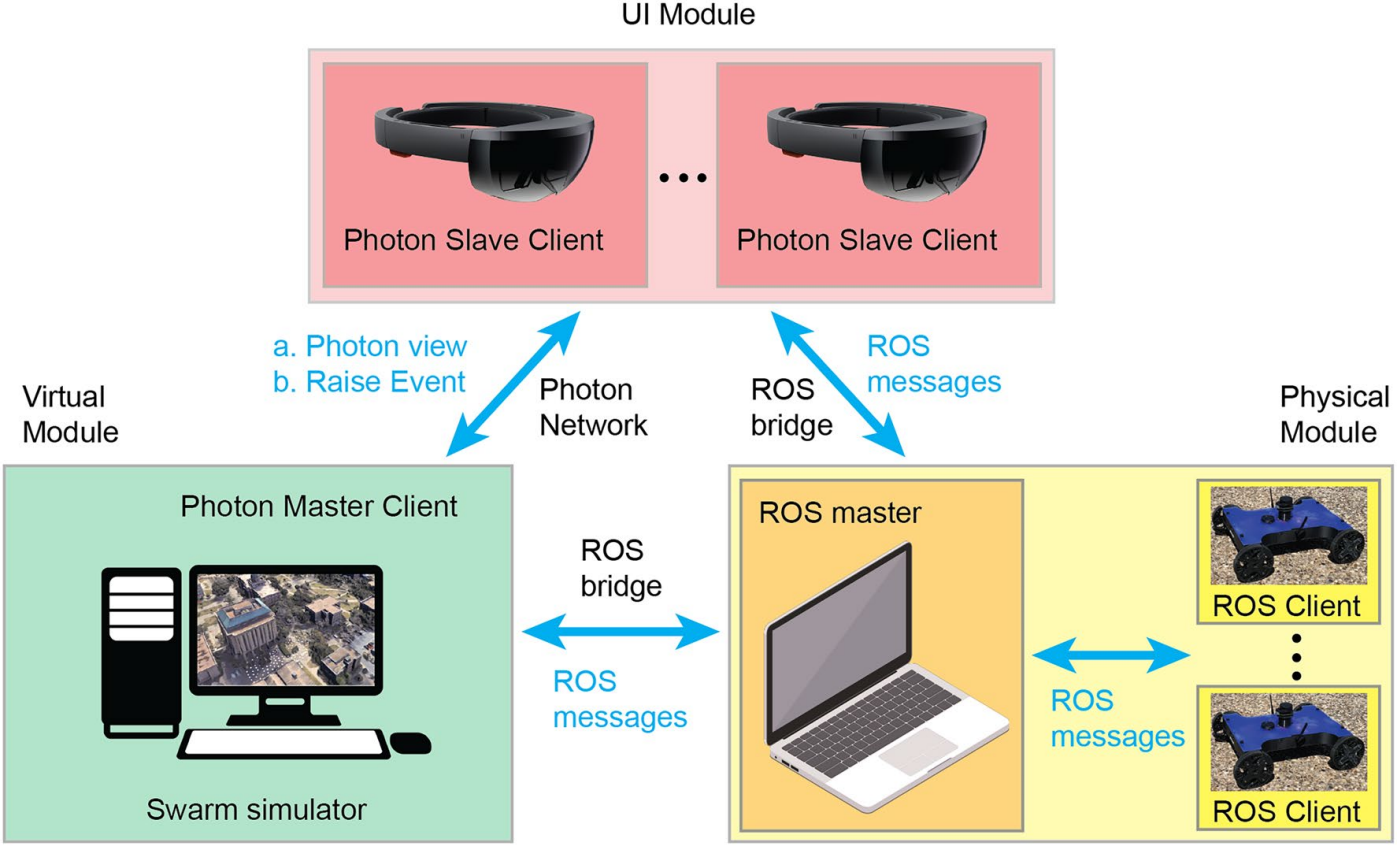
The HyRoS system architecture for the virtual-physical robotic swarm.

This section introduces the system architecture and technical components that underpin the HyRoS system and HSI. The integrated system comprises three key components: a virtual module, a physical module, and a UI module. The interconnection between these modules is illustrated in Fig. 1, demonstrating the integration facilitated by ROS bridges and the Photon network.

### Hybrid swarm system architecture

The virtual module contains the swarm simulator running all algorithms related to the control of virtual agents. The embedded include consensus decision-making, consensus-based task allocation, locomotion, collision avoidance, and swarm behavior algorithms. More details about these algorithms are provided in Section “Embedded swarm algorithms”. The UI module contains one or more UI devices, each used by a human user. Each user can act as a task planner who defines a task list using a sand table visualization or an observer to visualize the swarm in the real environment on a 1:1 scale. The UI module connects the virtual module through the Unity Photon network^39^, which enables the transmission of messages and synchronization of the robots’ positions, orientations, and scales. The Photon network allows multiple UI devices to connect to the same simulator and share information among all connected agents. The physical module runs the ROS network consisting of a ROS core and multiple ROS clients, each corresponding to a physical robot.

The connection between the virtual/UI module and the physical module is established through a ROS bridge, which allows the transmission of ROS messages. Employing the ROS-sharp package in Unity^40^, publishers and subscribers can be created with topic names that match certain topics in the physical module. Table 1 lists the topics transmitted through the ROS bridge. The UI devices not only send GPS goals and motion commands to the physical robots but can also adjust their speeds and trigger stair traversing functions when needed. On the other hand, the physical robots always share their GPS locations and heading angles with the swarm simulator and are ready to provide real-time camera views through the UI devices upon request. This connection enables virtual and physical agents to form a swarm and coexist in the real world, collectively executing swarm algorithms. A hybrid virtual-physical swarm is created by superimposing clone agents with synchronized poses of the physical robots in the simulator. If a clone agent is assigned a task, the information related to this task is sent to the corresponding physical agent through the network. The physical and UI modules are also connected through a ROS bridge, facilitating direct information exchange between the UI devices and the physical robots.

**Table 1:** ROS topics transmitted through ROS bridge.

Publisher	Subscriber	Description
UI	Physical	Send GPS goal location to physical robots
UI	Physical	Send motion commands to physical robots
UI	Physical	Adjust linear & rotation speed of physical robots
UI	Physical	Trigger stair traversing algorithm of physical robots
Physical	Virtual	Update GPS location of physical robots in the simulator
Physical	Virtual	Update heading of physical robots in the simulator
Physical	UI	View real-time camera view of physical robots

### HoloLens and Unity MR toolkit

HoloLens 2 (Microsoft Corp, Redmond, WA) was selected as the MR-based UI device. It is a pair of smart glasses embedded with multiple sensors, advanced optics, and a holographic processing unit. It has been used in a broad range of fields, including education^41, 42^, architecture^43, 44^, medicine^45, 46^ and industrial engineering^47, 48^. Unity is one of the leading real-time development platforms in the market, often used to develop MR-based applications. One easy way to develop an MR application is to use the *Mixed Reality Toolkit*^49^, which sets up a project automatically and provides a set of useful features^50^. Specifically, we adopted the following tools to accelerate the MR application development^51^:

- *Virtual hands:* Virtual left and right hands are available when an MR project runs in Unity. This feature enables simulating hand-based operations without wearing a HoloLens device.
- *Interactable objects:* Setting an in-game object to be *interactable* allows hand-based operations on this object, such as grabbing, moving, rotating, and scaling.
- *Buttons and menus:* Various types of buttons and menus are provided offering desired modes of control in different situations.
- *Hand tracking:* This feature allows recognizing simple hand gestures, such as pointing and air tapping, as well as customized hand gestures. Hand tracking is realized by detecting the positions of 25 joints on each hand^52^.
- *Eye tracking and voice commands:* Eye tracking and voice commands are also available.

### Virtual environments and spatial anchors

**Figure 2:**
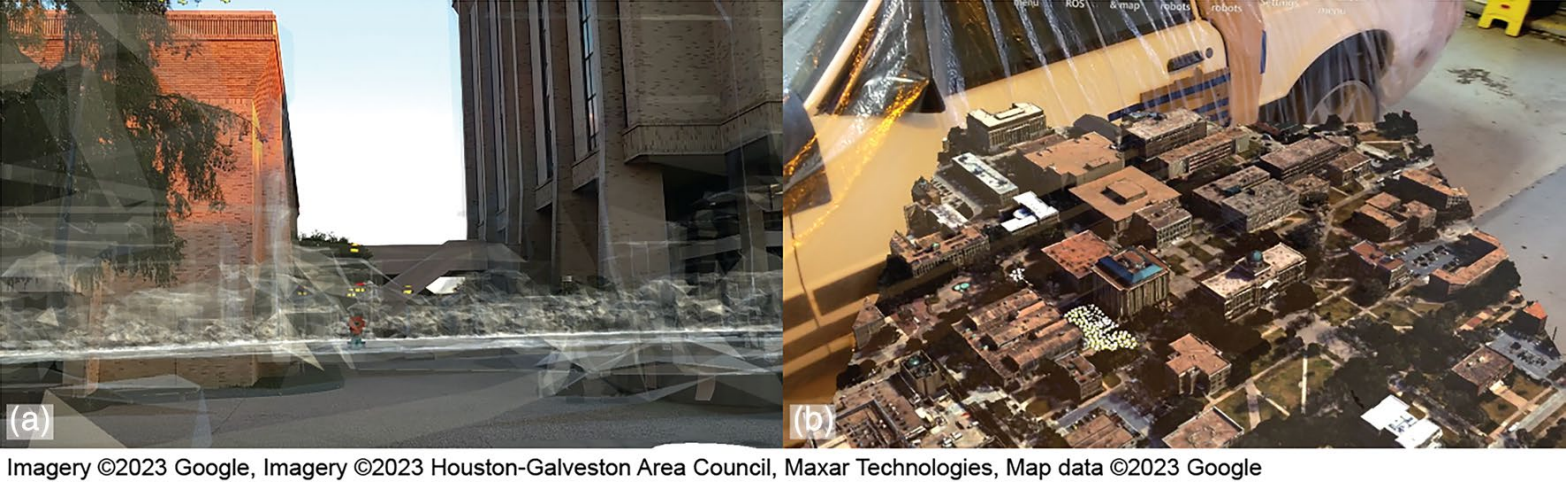
Virtual map: (**a**) overlaid on the real environment, and (**b**) miniaturized and displayed in front of the user.

**Figure 3:**
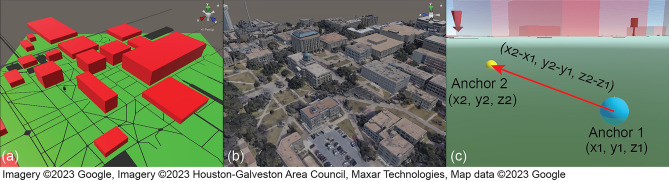
TAMU campus map in Unity sourced from (**a**) OpenStreetMap, and (**b**) Google Map, and (**c**) spatial anchors.

MR merges the real and virtual worlds to produce a new visualization. While virtual agents may take on any designs or forms, the virtual and real maps should closely match with each other to allow proper visualization of the operating environment and performance of the robotic agents. By matching the virtual map with the real environment, the simulated movements of virtual robots can be visualized in the real environment as if they were real. The virtual map can overlay the real environment in a 1:1 scale view or can be miniaturized and displayed in front of the user in a virtual sand table (See Fig. 2). The virtual 3D representation of the environment may be created in two ways. A simplified map for a target operation location can be sourced from *OpenStreetMap*^53^ (Fig. 3a) and converted to a Unity-friendly format with the following steps:

- *Source from OpenStreetMap:* Select the boundary of the map and export the data in ‘.osm’ format.
- *Format conversion:* Convert the map data from ‘.osm’ format to ‘.obj’ three-dimensional models with *OSM2WORLD*^54^ application.

A high-quality map (Fig. 3b) can be sourced from *Google Maps*^55^ and recreated in Unity with the following steps:

- *Source from Google: Map* Select the boundary of the map and export the data in ‘.rdc’ format utilizing the *Renderdoc*^56^ application.
- *Format conversion:* Convert the map data from ‘.odc’ format to ‘.obj’ three-dimensional models with *Blender* software.

After converting the map data to ‘.obj’ 3D models, the map is ready for use in Unity. 3D maps generated by Google Maps are based on real satellite images and are thus more detailed and realistic. However, they are generated from previously collected images, and thus some inherent differences exist between the 3D map and the real environment. However, the outlines and locations of buildings and other fixtures remain unchanged.

A spatial anchor functions as a virtual flag, which can be placed in the real world to mark a location of interest. As a default option, the Azure spatial anchor^57^ is available. Azure is a cloud service that allows uploading spatial anchors to enable multi-user and cross-platform applications. However, it is sensitive to changes in the physical environment and highly dependent on the stability of the cloud server and internet connection. To address these limitations, a customized spatial anchor system was created to replace the Azure service, as shown in Fig. 3c. This anchor system contains two individual anchors: Anchor 1 shown in blue, and Anchor 2 shown in yellow. Anchor 1 determines the position of the map. The vector from Anchor 1 to Anchor 2 determines the map’s orientation. After marking the two positions in the real environment and placing the two anchors properly on the marks, the position and orientation of the map are calibrated to match the real environment. This process can be repeated to accurately align the virtual and real environments.

### Ethics approval and consent to participate

This research does not involve any Human Participants and/or Animals.

## Embedded Swarm Algorithms

The swarm simulation system has embedded consensus decision-making and task allocation algorithms for executing a list of tasks. The tasks may be created by a human operator or autonomously if the swarm is equipped with such capabilities. This section reviews the swarm consensus algorithm, task allocation strategy based on consensus decision-making, and embedded swarm algorithms used for our demonstrative experiments.

### Review of swarm consensus decision-making^58^

Consensus decision-making is considered one of the fundamental capabilities of a swarm to perform a task collectively. While the embedded consensus algorithm was previously presented in^58^, it is briefly revisited here to better explain the embedded task allocation strategy based on this algorithm. In our swarm system framework, each agent is assumed to have *N* embedded *swarm algorithms* while *N* may change as the agent learns/receives new algorithms or deletes unnecessary ones. Individual agents execute one algorithm at a time. Collective swarm behavior can be achieved when all or many agents execute the same algorithms simultaneously. The agents must achieve consensus through distributed, localized communication to do so. A swarm consisting of *n* agents is denoted as ${\mathcal {S}} = \{A_1, A_2, \ldots , A_n\}.$ Each agent has *N* embedded swarm algorithms, ${\mathcal {Q}} = \{q_1, q_2, \ldots , q_N\}.$ Neighbors of $A_k$ include the agents within the communication range of $A_k$, such that ${\mathcal {N}}_k = \{N^k_1, N^k_2, \ldots , N^k_r \}$. The individual agent’s preference toward *N* possible options is modeled as a probabilistic mass function (PMF). The index of the most preferred option $D_k = index(\max$ $\{p^k_1, p^k_2, \ldots , p^k_N\})$ is the exhibited decision or state of $A_k$.

The consensus algorithm is based on each agent’s exhibited decision $D_k$ and the level of certainty defined as $\Omega _k = {1}/(H_k+1)$, where $H_k$ is the discrete entropy on $A_k$’s preference distribution, such that $H_k = -\sum _{i=1}^{N} p^k_i \log _2 p^k_i$. For *N* available options, ${1}/(1+\log _2 N) \le \Omega \le 1.$ Local negotiation involves each agent exchanging limited information with its neighbors. In our work, this involves the index of the most preferred option and the level of certainty, such that

1 $$\begin{aligned} {\vec {p}}_k^{new} = \left[ \frac{{\vec {p}}_k + \sum _{i=1}^{r} \lambda ^k_i\vec {\Omega }_{N^k_i}}{\sum _{j=1}^{N}\left( {\vec {p}}_k(j) + \sum _{i=1}^{r}\lambda ^k_i\vec {\Omega }_{N^k_i}(j) \right) } \right] \end{aligned}$$

where $\vec {\Omega }_{N^k_i}$ is an *N*-by-1 vector with $\Omega ^k_i$ on the $j$th element where *j* is the index of the exhibited decision of $N^k_i$, i.e., the $i$th neighbor of $A_k$, and zeros for the rest. $\lambda ^k_i$ is a weight that may be constant across all agents or individually assigned. Considering $0<\Omega _{N_i^k}\le 1$, we set $\lambda _i^k \le 0.5$; thus, the agent takes account for its own preference and the neighbors’ opinions in a balanced manner. In the presented work, we used $\lambda = 0.3.$

### Task allocation and task re-prioritization

We implemented the consensus algorithm for swarm task allocation. Considering a swarm of robotic agents, we assume that an upper-level task list is either sent to a subset of the swarm, and the swarm forms a task group for each task autonomously via localized communication and consensus decision-making. Two types of information are shared through local communication for task allocation: *task list* and *task group*. A task list contains one or more tasks to be performed by the swarm. Task group information contains information on the number of locally converged agents who exhibit the same task decision. Unlike the task list remaining unchanged and shared across the swarm, task groups continuously change as the exhibited decisions of the agents evolve until the swarm reaches consensus.

A task list may involve one or more tasks, each specified by the task type, agent type, priority, location (specified by the GPS coordinate), time duration, and workload (i.e., the number of robotic agents required for the task). Within the scope of the presented work, the task type indicates the target swarm behavior that can be achieved by the agents executing embedded swarm algorithms. The agent type specifies what kind of robots (e.g., UAV or UGV) is needed for the task. The priority number sets the relative importance of the task, and we used a 1 (low)–5 (high) scale. In the presented system, the user specifies the task-specific information and creates a list. This operation may be replaced by autonomous algorithms. For example, once the user selects the desired tactic (e.g., loop formation) and specifies the target location, an embedded algorithm can autonomously calculate the number and type of agents needed to complete the task. Task allocation follows the steps: (1) a task list is sent to a specific agent or a subset of agents and shared with the entire swarm via local communication; (2) the agent closest to one of the task locations initializes consensus to form a task group; (3) unassigned agents are recruited to join one of the task groups via the consensus protocol; and (4) individual agents terminate the consensus process when the number of task group members reaches the workload.

Task re-prioritization allows individual agents or a subset of the task group to pause their current task and participate in a different task with a higher priority. Given any new task with a higher priority, the agents may be reassigned to the new task; upon completing the newly assigned task, they may return to the previous task. Currently, the system has a default setting of priority 2 for all tactics other than target tracking. The target tracking tactic has priority 3, which is higher than the rest tactics. If necessary, a tuning option for the priority of tasks can be added to the UI, so that users can easily change the priority of each task. This scheme is utilized in the system demonstration (Demo 1) and detailed in Section “Demonstrations”. It is important to highlight that the task allocation approach employed in this work is a consensus-based algorithm, where individual agents make decisions based on their own preferences and requirements. In this particular implementation, the distances from each robot’s current location to the task locations were taken into account, giving priority to tasks located closer to the robots. While the global optimality of this task allocation scheme is not evaluated in this context, it is worth noting that achieving global optimality in a distributed system can be challenging and depends on specific optimization criteria.

### Algorithms for collective swarm behavior

Within the presented framework, each robotic agent may have several embedded swarm algorithms corresponding to collective swarm behaviors. Specific algorithms may be defined for each target application. The embedded consensus and task allocation algorithms allow any number/type of swarm algorithms to be considered. For system-level demonstrations, we considered four specific algorithms, including $\mathcal {Q}=\{(q_1: \text {aggregation}), (q_2: \text {dispersion}), (q_3: \text {loop formation}), (q_4: \text {target tracking})\}$. Individual algorithms are considered relatively simple, while the corresponding swarm behavior is carried out by a group of agents executing the same swarm algorithm together. We employed simple strategies to achieve these four collective swarm behaviors based on local interaction and communication, as described below. We note that other swarm algorithms can replace these, or new ones may be added.

The aggregation tactic (Algorithm 1, $q_1$) aims for the swarm to collectively move to a target location and gather closely at that location. This is achieved by forming multiple layers of circular rings around the target point. During this tactic, the agents move toward the target and occupy the rings. Starting from the inner ring, each agent moves in the counterclockwise direction on the ring until it is close enough to a neighbor on the same ring. After a ring is out of available space, the agents occupy the outer ring next to it, following the same rule. The aggregation tactic is accomplished after all agents gather close to the target location, where each individual stays on one of the rings.

The dispersion tactic (Algorithm 2, $q_2$) is used to cover an area with swarm agents. To proceed with the dispersion tactic, the swarm performs aggregation at the target point first and then disperses by spreading out until it reaches the desired distance. Based on a “spring-damper model”^59, 60^, the agents continuously push each other along the direction of the combined force of the three closest neighbors until the force is smaller than a certain threshold.

The tactic for loop formation (Algorithm 3, $q_3$) enables the swarm to form a loop around the target position. In this tactic, the swarm aggregates at the target point first and then spreads out until the agents reach the circle boundary. Each agent then moves either in the clockwise or opposite direction along the boundary while keeping the number of neighbors equivalent on its left and right sides. This rule guarantees an even distribution of the agents on the circle boundary.

The target tracking tactic (Algorithm 4, $q_4$) requires a fixed number of agents to track and follow a moving target. Whenever a moving target appears within a detectable range, the agent(s) triggers the consensus process to recruit other agents and form a task group. In the presented work, five agents were required to form a task group corresponding to $q_4$. Based on the IDs of the agents, each is given a specific relative position with respect to the moving target. While the agents track the target, they send real-time camera views of the target to the operator(s) until they receive a feedback command by the operator(s). If “Disable Target” is received, the agents eliminate the target. If “Ignore Target” is received, the agents leave the target and resume their previous task.


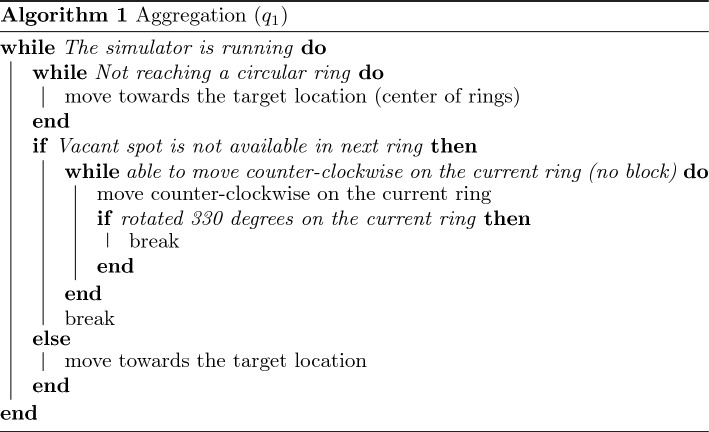



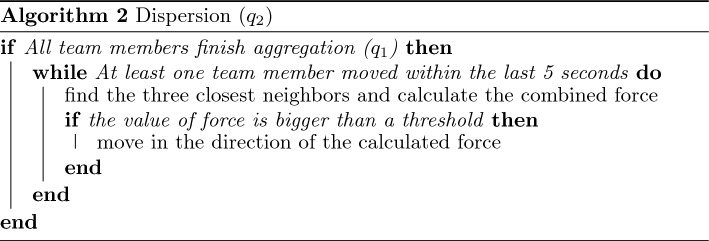



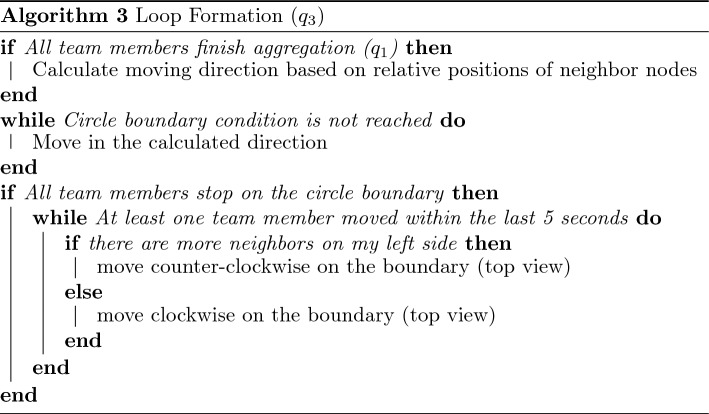



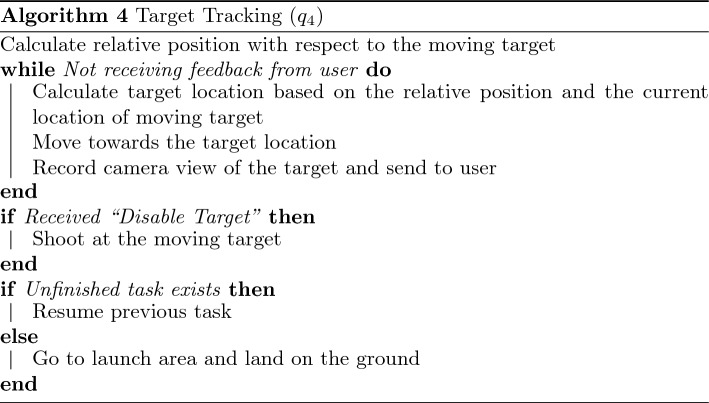


## Hybrid swarm system and user interface

This section introduces the HyRoS system to support different compositions of virtual and physical robotic agents and the UI design to facilitate multi-user roles and levels of interaction.

**Figure 4:**
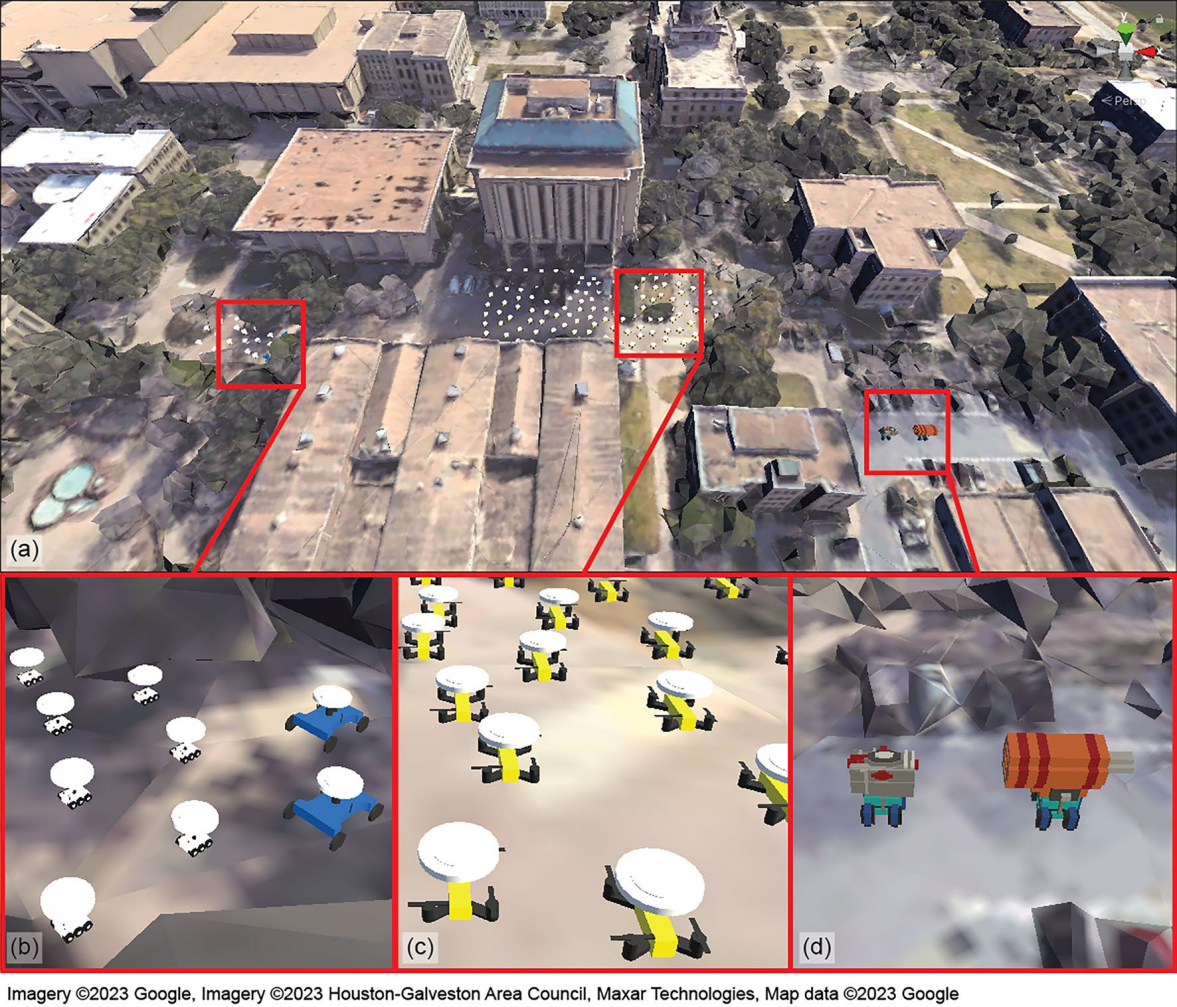
Virtual-physical hybrid swarm simulator: (**a**) virtual map, (**b**) virtual swarm of UGVs (white) and clones of physical UGVs (blue), (**c**) virtual swarm of UAVs, and (**d**) moving targets.

### Virtual-physical hybrid swarm simulator

For virtual-physical hybrid simulations, we must first select the physical environment where the system operates. Once the site is determined, a virtual representation of the environment is created as the digital counterpart of the real world. Our test environment was located on the Texas A&M University campus in College Station, Texas, USA, with the GPS coordinate (30.6169, − 96.3410). We followed the steps described in Section “Virtual environments and spatial anchors” to import the 3D map from Google Maps. Fig. 4a shows the virtual environment created in Unity. The quad around the center of the map serves as the launch area where all UAV agents are initially located and the area on the left is the initial location of the UGV agents.

Figure 4b shows the UGV models, and Fig. 4c shows the UAV models used in the simulations. The floating circles on the top of each agent visualize the task status and priority of the current task using different colors. White indicates no task assigned to the agent. Figure 4d shows two moving targets, which do not belong to the swarm. In Fig. 4b, two ground agents shown in blue are the clones of the physical robots, each projecting a specific physical robot. The location and orientation of each clone agent are updated in real-time to match the corresponding information of the physical robot. Having the same communication and consensus decision-making capabilities as the virtual agents, the clone agents participate in the task allocation process and execute the assigned tasks with virtual agents. Each agent is programmed to have the following capabilities:

- *Locomotion:* It can move freely within the environment while avoiding obstacles.
- *Collision avoidance:* It avoids collision with other agents using a predefined collision boundary.
- *Localized peer-to-peer communication:* Each robot can communicate with its neighbors within the defined local communication range.
- *Consensus decision-making:* Each agent can participate in consensus decision-making to allocate tasks to the swarm autonomously.
- *Swarm algorithms:* Each UAV agent is embedded with a default set of swarm formation control algorithms, including $\mathcal {Q}=\{(q_1: \text {aggregation}), (q_2: \text {dispersion}), (q_3: \text {loop formation}), (q_4: \text {target tracking})\}$, and each UGV agent is embedded with only one algorithm corresponding to $\{(q_1: \text {aggregation})\}$ within the scope of the current work.

**Figure 5:**
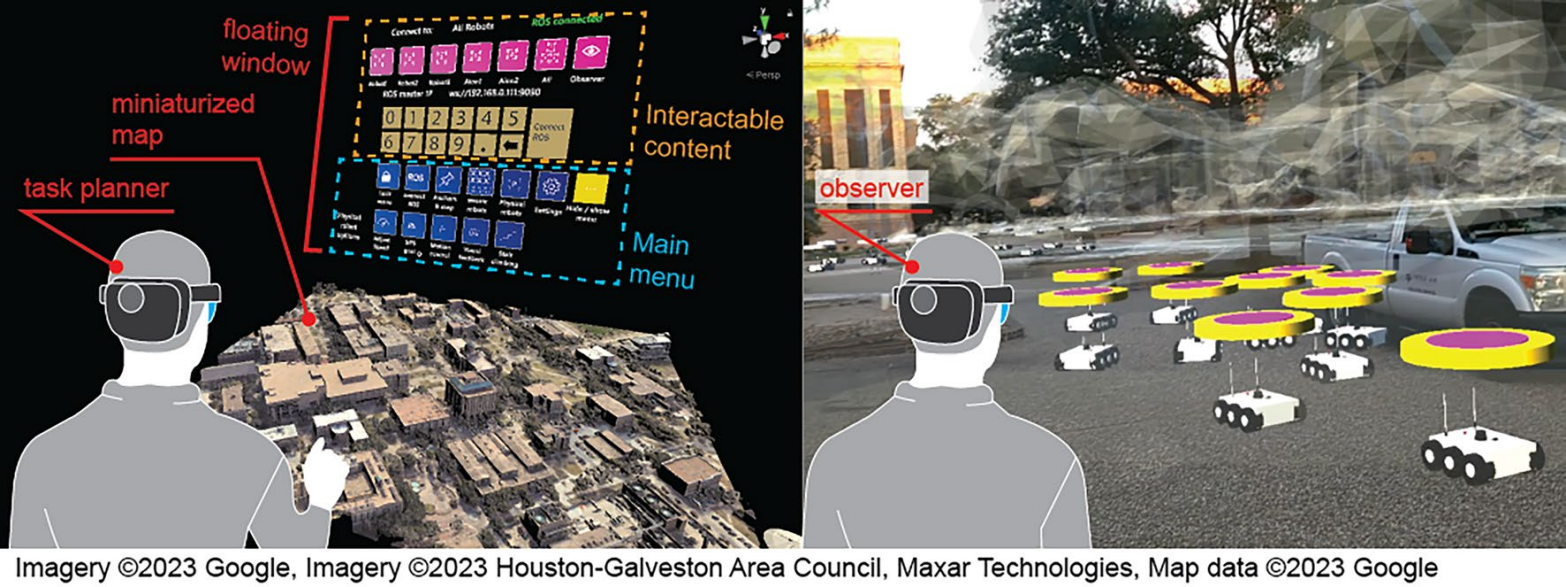
Human–swarm interface: the task planner’s view with miniaturized map (left) and the observer’s view with 1:1 map (right).

### MR-based UI design

The MR-based UI was designed to support (1) a task planner operation with a view of a miniaturized 3D map (Fig. 5 (left)) and (2) an observer or direct control operation with a 1:1 scale view of the MR, i.e., virtual agents and map overlaid on the real environment (Fig. 5 (right)). For task planning, the map functions as a sand table. A bird’s eye view of the mini-map enables the user to easily define the task locations and visualize the overall distribution and activities of the swarm agents. The 1:1 interaction mode visualizes the movements of the virtual agents in the real environment as if they were real robots. The system allows the user to switch between the two modes efficiently using the floating window, which provides a complete set of interactive content to realize the interaction between the user and the hybrid swarm. The floating window includes the following tabs:

- *Connect ROS:* The user connects the HoloLens to the ROS system by inputting the IP address of the ROS master.
- *Anchor & map:* The user calibrates the virtual map by either inputting the GPS location of the user’s standing point or dragging the spatial anchors to predefined real-world marks. The user may switch the map between miniaturized and 1:1 scale versions.
- *Task planning:* The user defines a list of tasks, each including a tactic, task duration, workload (number of swarm agents needed), and task location. The user may also activate a certain moving target.
- *Physical robots:* The user interacts with one or multiple physical robots in the following aspects: speed adjustment, GPS goal input, gesture control, camera view visualization, and stair-climbing command.
- *Settings:* The user tunes the visual settings, such as the scale of UGVs & UAVs and animation of propellers.

**Figure 6:**
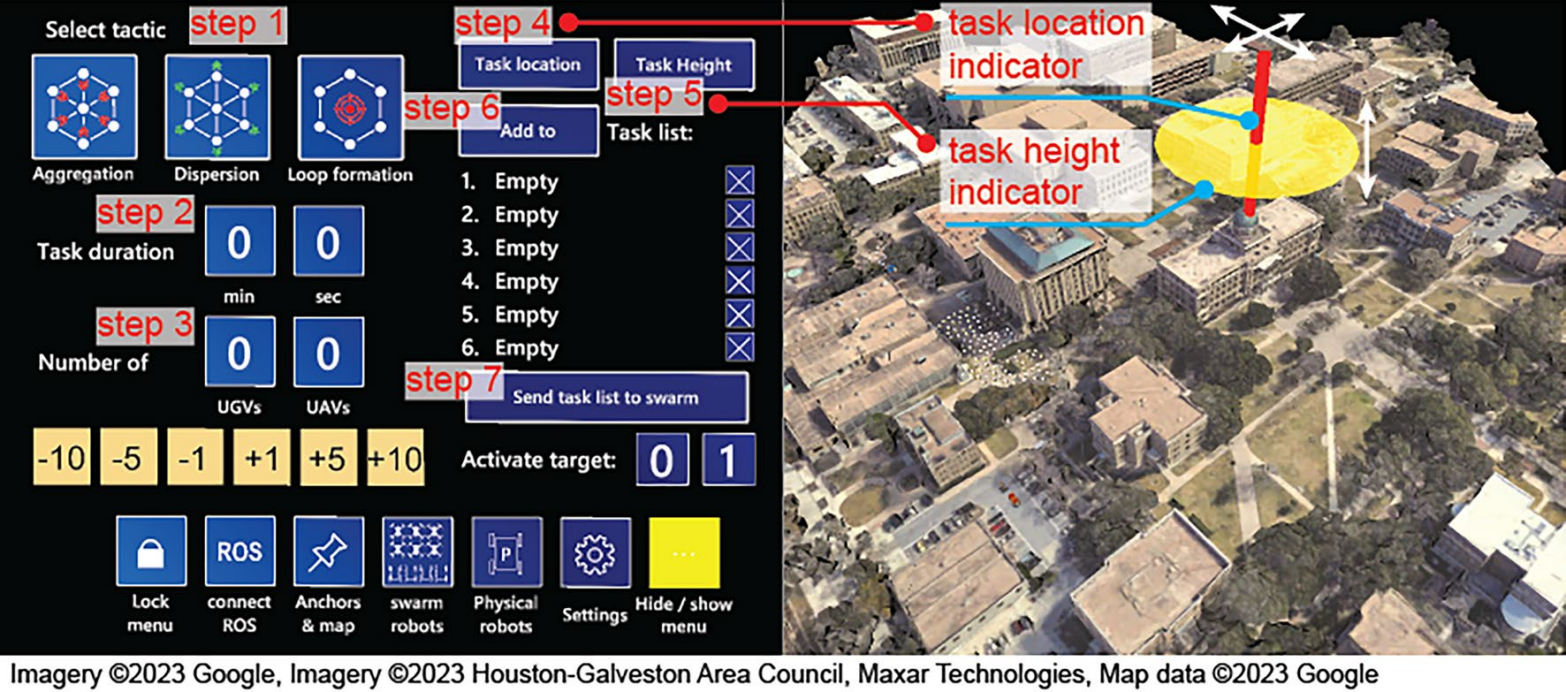
Steps to define and send a task list to the swarm.

Before defining a list of tasks, a user must set the map to the miniaturized version. Upon clicking “Task planning” on the main menu, the window shown in Fig. 6 opens up for defining a task list by following the steps below:

1. *Select tactic:* The user selects one of the three following tactics, Aggregation, Dispersion, and Loop Formation.
2. *Set task duration:* The user defines the time duration of the current task.
3. *Set workload:* The user defines the number UGVs or UAVs needed for the current task.
4. *Set task location:* The user defines the GPS location of the current task by dragging the task location indicator on the miniaturized map.
5. *Set task height:* The user defines the height of the current task by dragging the task height indicator on the miniaturized map.
6. *Add to task list:* The user adds the newly defined task to the task list.
7. *Send task list:* The user repeats the previous steps to define and add each new task to the list. Once all tasks are defined, the user sends the task list to the swarm.

The system can support on-the-fly modifications of tasks by adding relevant functions at the programming level. The user may initiate an emergency stop command directed toward a specific task group followed by reassigning a modified task. When a significant change is required across the entire swarm, all task groups may be recalled for task re-allocation. If frequent on-the-fly operations are expected, these functions can be embedded into the UI.

## System demonstrations

This section focuses on technical evaluations of (1) the communication latency and (2) system-level integration via two demonstrative experiments.

### Technical evaluation of communication latency

For the HyRoS system to serve as a swarm simulation tool and offer interactive UI modality, the system must show minimal communication latency among the system modules (i.e., UI, virtual, and physical modules in Fig. 1). The communication latency is influenced by the wireless communication devices and protocols employed to connect the system modules. Even when using the same wireless communication method, the communication speed and quality can be significantly impacted by various internal and external factors. In our current system setting, we used the TP-Link AX6000 router for establishing the local wireless network.

To quantitatively evaluate the latency between the UI module and the other two modules, the time required for a small packet with the number “1” to travel back and forth was measured 30 times. The result shows that the average time for a round-trip was 0.600 second with a standard deviation (SD) of 0.020 between the UI module and the virtual module. It was 0.040 second (SD = 0.007) between the UI module and the physical module. The average one-way communication times were 0.300 and 0.020, respectively. The UI and the virtual modules communicate through the Photon network, which creates a cloud room for all clients to connect through the internet; on the other hand, the UI module connects to the physical module under the ROS system directly through a local wireless network. Therefore, the communication between the UI and the virtual modules takes significantly longer than between the UI and the physical modules. A previous study found no significant impact of the latency of up to several seconds on user performance in multi-player, real-time strategic games^61^. The communication time among the modules varies based on the types and size of data being exchanged. Nevertheless, the controlled experiments carried out in this study serve as a technical baseline. To reduce latency when using the Photon Network, switching to a local server instead of relying on a cloud-based one could be advantageous. Moreover, upgrading to a wireless communication device with a higher frequency and wider bandwidth has the potential to significantly enhance the system’s overall performance.

### Demonstrations

To demonstrate the system-level integration of the proposed hybrid MR-based UI and technical functionality, we designed two experiments: (1) involving two users (a planner and an observer) and a swarm of 170 virtual agents (20 UGVs and 150 UAVs), and (2) involving a single user and a hybrid swarm of 170 virtual agents and two physical robots. In Demo 1, the planner creates a task list, and the swarm autonomously forms task groups and executes the task upon receipt of the list. In Demo 2, the planner assigns a similar task list for the swarm to perform, while also involving direct control of a physical robot to complete the task. Detailed descriptions and results are presented below.

**Figure 7:**
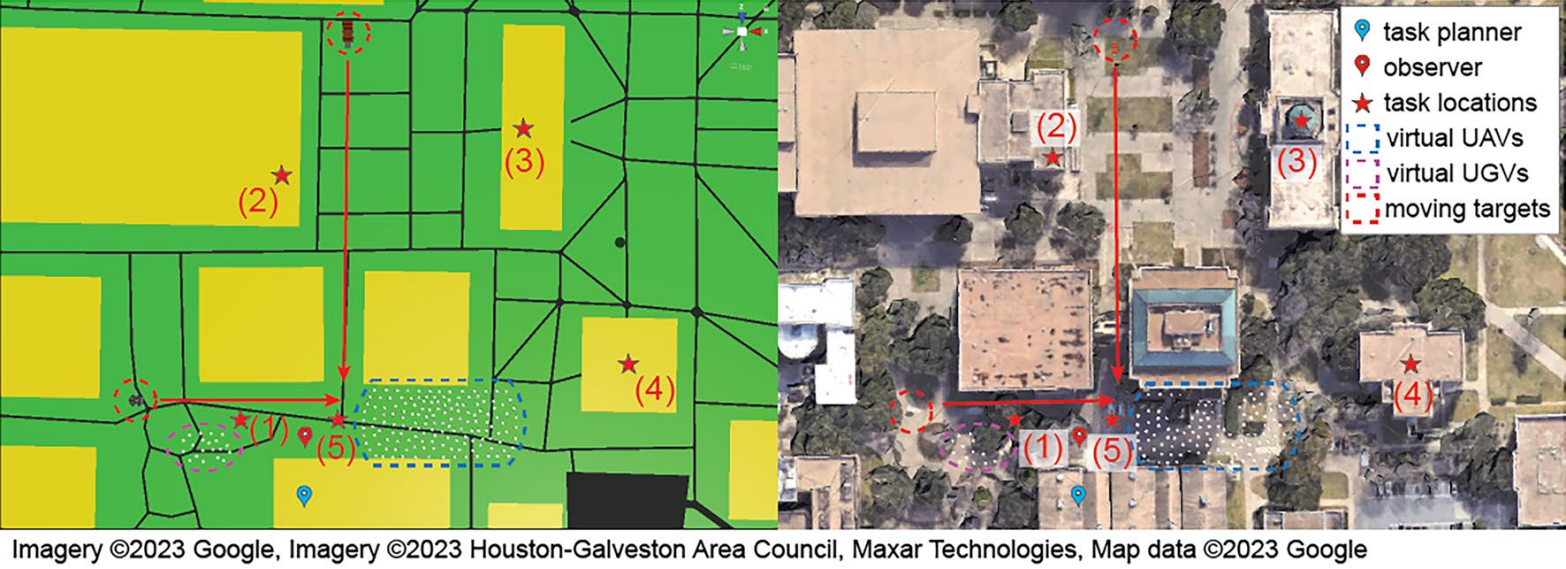
Storyboard of experiment 1 in a simplified visualization (left) and the aerial Google map view (right).

**Table 2:** Task list in experiment 1.

Index	Task Type	Agent	Priority	Location	Duration	Workload
1	$q_3$: Loop formation	UAV	2	(1)	600	20
2	$q_3$: Loop formation	UAV	2	(2)	600	20
3	$q_1$: Aggregation	UAV	2	(3)	300	20
4	$q_2$: Dispersion	UAV	2	(4)	300	40
5	$q_1$: Aggregation	UGV	2	(5)	Null	10

Locations as shown in Fig. 7 and Duration in seconds.

#### Demo 1: task allocation of the virtual swarm

**Figure 8:**
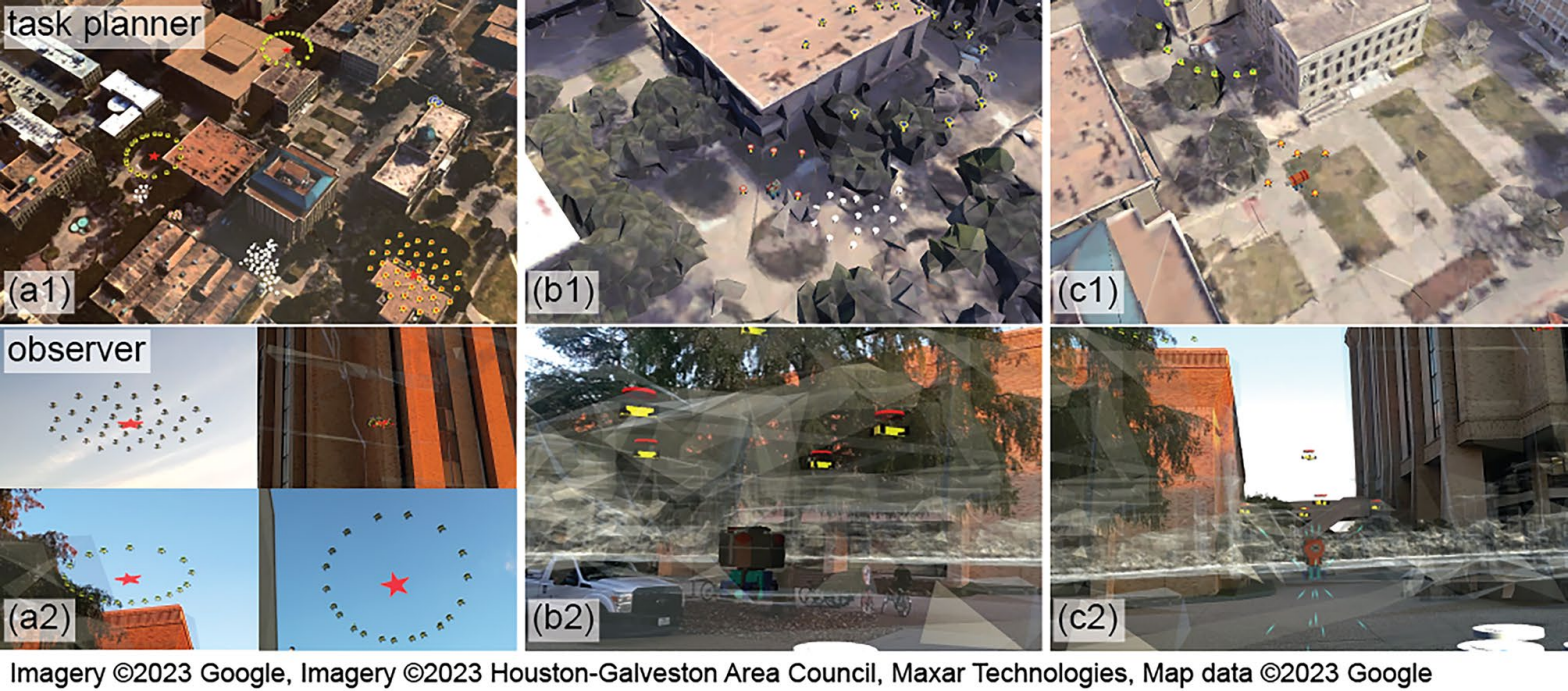
Experiment 1: the task planner’s view (top) and the observer’s view (bottom).

Demo 1 involved two users playing the roles of a swarm planner and an observer. Their physical locations are marked with the blue and red pins in Fig. 7. The planner creates a list of tasks by specifying the task type, priority, robot types, location, time duration, and workload for each task. Table. 2 shows the task list to be defined by the task planner and sent to the swarm in this demonstration. Tasks 1–4 were for UAVs and Task 5 for UGVs, where red stars in Fig. 7 indicate the task locations. Upon receiving the task list, the task allocation function autonomously formed five task groups, one for each task. The number of agents within each group matched the workloads defined in the task list. Upon forming the task groups, each group departed for the corresponding task location and executed the task successfully. Figure 8 shows the planner and observer views of the swarm activities. Figure 8a shows the two user views of the task groups at individual task locations.

**Figure 9:**
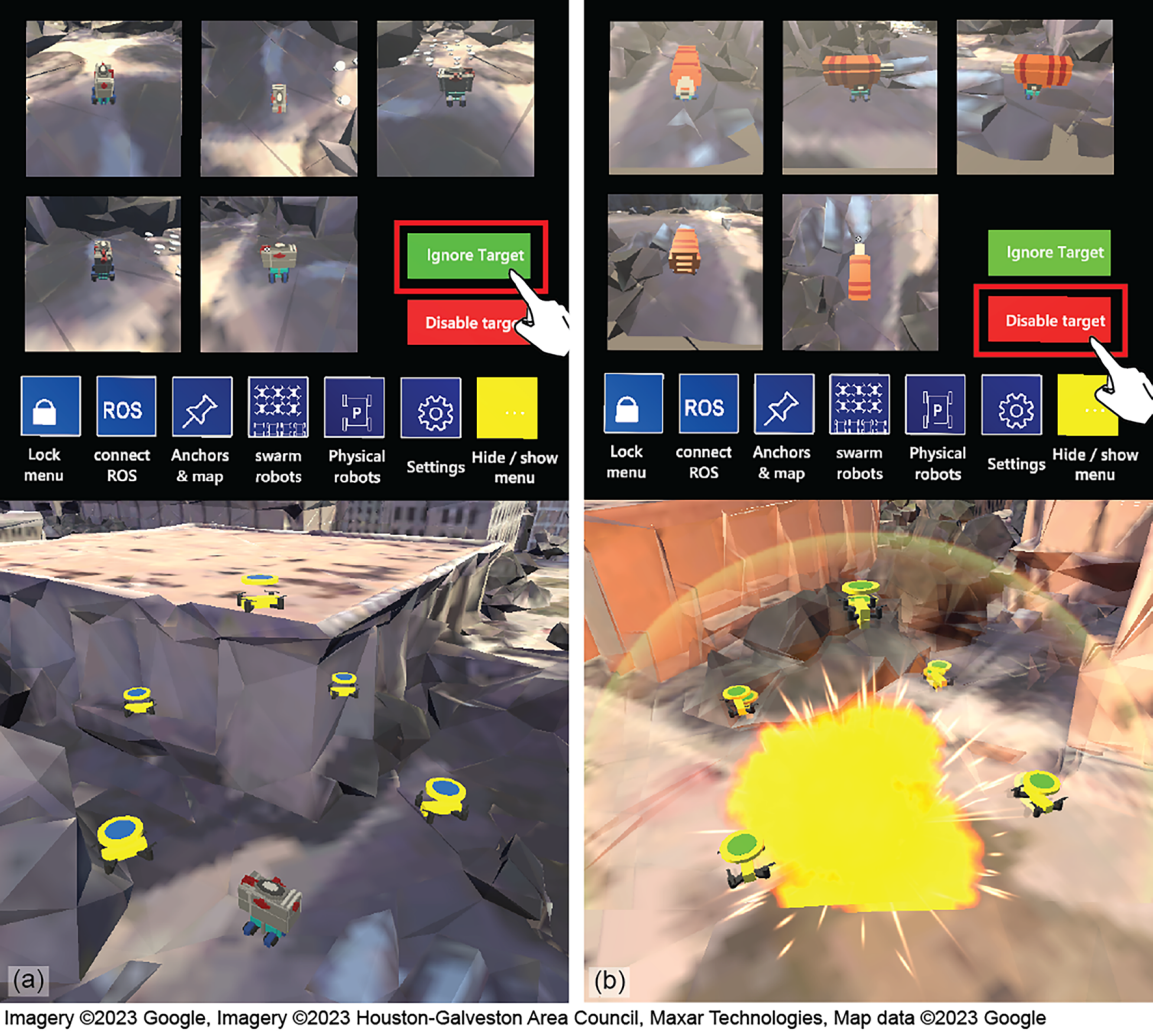
Real-time video feed provided to the user when an abnormal activity (i.e., a moving target) is detected.

While the task groups performed the assigned tasks, two mobile targets were separately activated to move in their predefined trajectories. As a mobile target approached the Task 1 location, the closest agent in Task Group 1 detected this target and triggered the consensus process to form a group of five agents to perform the target tracking task (Fig. 8b), which has a higher priority than the task currently being performed. These agents activated the real-time camera views for the planner to inspect the target, as shown in Fig. 9. After visual inspection, the planner determined that the target was not a threat and clicked the “Ignore” button (Fig. 9a). The five agents then returned to the previously assigned task location. The second target passing through the Task 2 location also triggered five UAVs to track and send camera views to the planner. The planner identified the second target as a threat and clicked the “Disable” button (Figs. 8c,  9b).

#### Demo 2: task allocation of the hybrid swarm and investigation of a target on the second floor

This experiment was designed to demonstrate the hybrid swarm capabilities, involving a single user and a virtual-real hybrid swarm consisting of two real UGVs and 170 virtual agents (20 UGVs and 150 UAVs). We used two α-WaLTR platforms^62, 63^ as the real agents. α-WaLTR is equipped with passively transformable wheels allowing the robot to climb over obstacles and staircases. Embedded with a Jetson TX2, 2 RGB-D cameras, IMU, GPS, and a 2D laser scanner, α-WaLTR can autonomously navigate in diverse environments.

**Figure 10:**
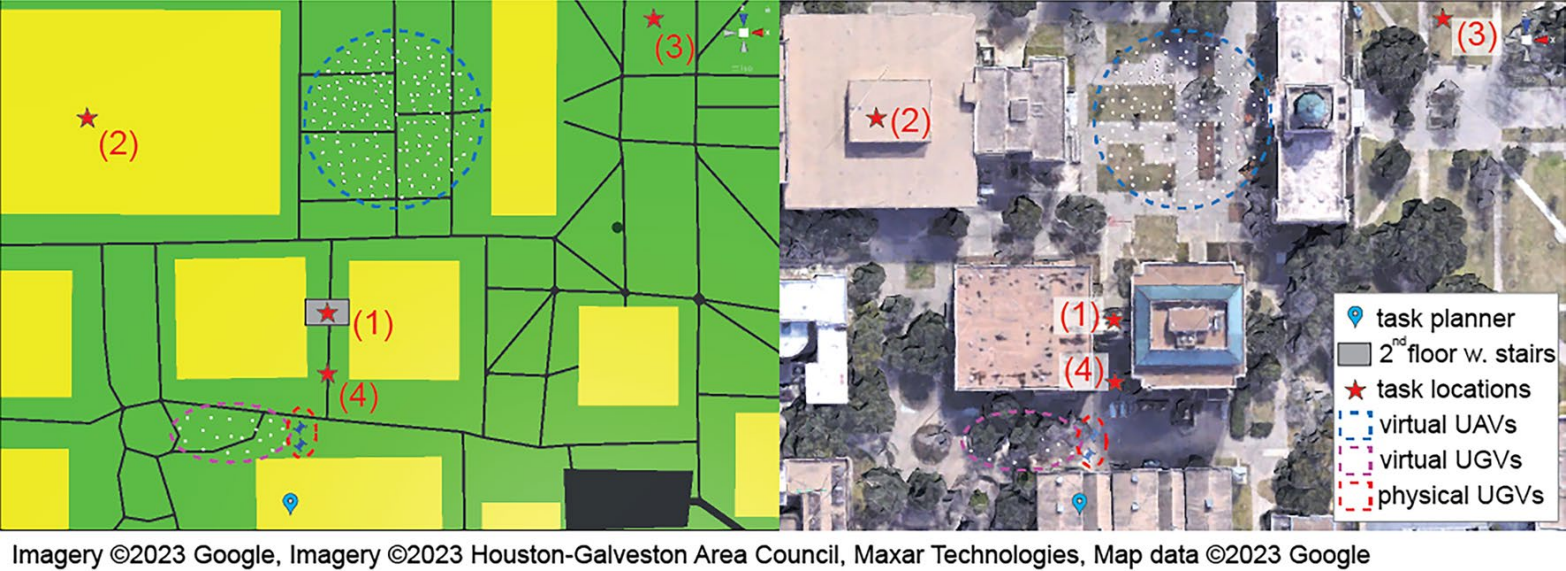
Storyboard of experiment 2.

**Figure 11:**
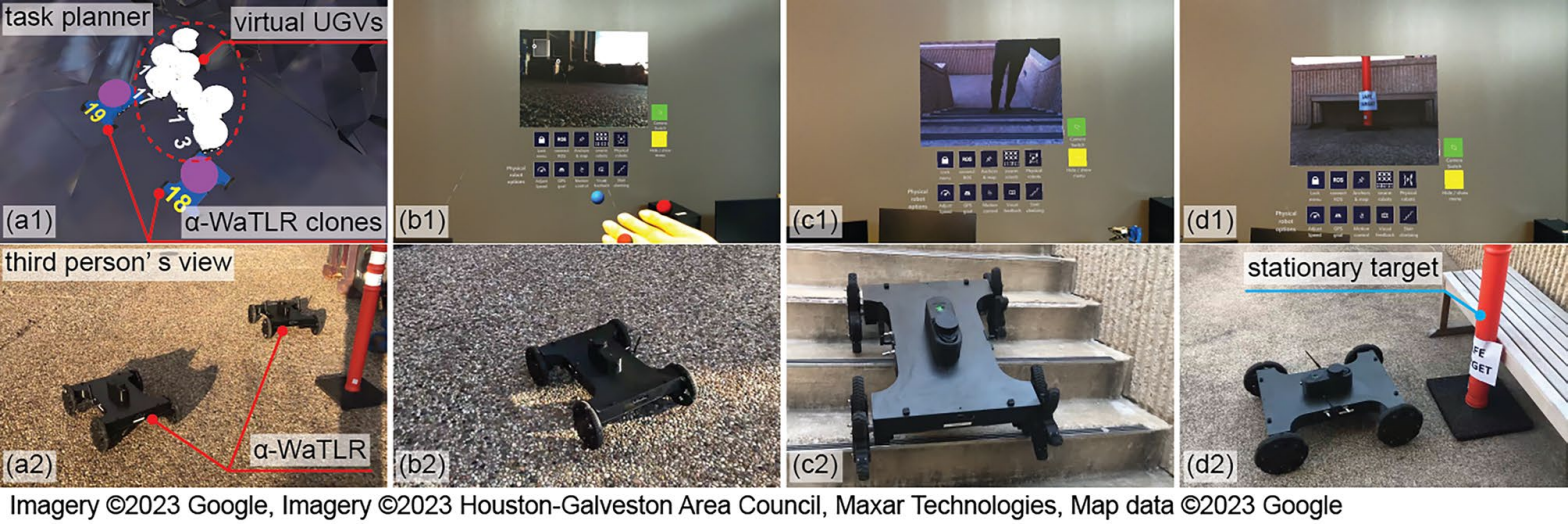
Experiment 2: the task planner’s view (top) and the third person’s view (bottom).

In this demo, the user created the task list with four tasks, as specified in Table 3. The task locations are marked with red stars in the storyboard shown in Fig. 10. After the task list was defined and sent to the swarm, ten UGV agents, including two α-WaLTRs were assigned to Task 4 and moved to the task location. Fig. 11a shows the planner’s and a third person’s views of the UGVs moving toward the Task 4 location.

**Table 3:** Task list in experiment 2.

Index	Task type	Type	Priority	Location	Duration (s)	Workload
1	$q_3$: Loop formation	UAV	2	(1)	600	20
2	$q_2$: Dispersion	UAV	2	(2)	600	40
3	$q_1$: Aggregation	UAV	2	(3)	600	20
4	$q_1$: Aggregation	UGV	2	(4)	Null	10

**Figure 12:**
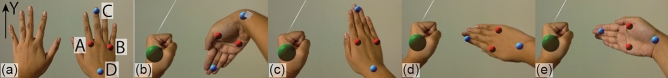
Customized hand gestures: (**a**) Emergency stop, (**b**) Move forward, (**c**) Move backward, (**d**) Turn left, (**e**) Turn right.


To demonstrate different levels of user interaction, this experiment also required the user to control one of the real robots via real-time gesture-based teleoperation. After the UGVs arrived at the Task 4 location, the user was tasked to inspect the second floor and search for a stationary target. Since α-WaLTRs can climb over stairs, the user selected one of α-WaLTRs to complete this task. Using the customized hand gestures defined in Fig. 12, the user operated the robot based on the real-time video feed. Algorithm 5 shows how to control a UGV using hand gestures by detecting the left fist and four joints on the right hand: IndexKnuckle (A), PinkyKnuckle (B), MiddleTip (C), and Wrist (D). Gestures corresponding to stopping, moving forward, moving backward, turning left, and turning right are shown in Fig. 12a–e. Once the robot reached the entrance of the staircase, the user triggered the stair traversing function^63^ and the robot climbed over the staircases using the embedded algorithms. After reaching the second floor, the user-controlled the robot using hand gestures to find the target. After inspecting the target, the user triggered the stair-traversing algorithm once again, allowing the robot to move down the staircases. The process of the direct control of α-WaLTR is shown in Fig. 11b–d. The supplementary media shows the recorded videos from the two demonstrations.

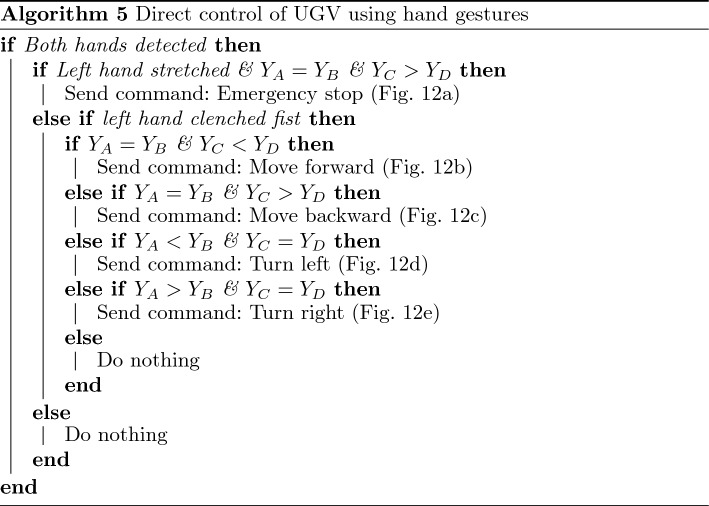


## Conclusion and discussion

The paper introduced an integrated system architecture for simulating virtual-physical hybrid swarms. The UI of the system was interfaced with the virtual component via the Unity Photon Network and with the physical component through the ROS system network. The MR-based UI allowed a user to define and send task lists to the swarm intuitively while also providing 1:1 control of individual robots through customized gestures and real-scale visualization. Two demonstrative experiments showed the successful integration of technical functions for hybrid swarm operations and different user roles. Future work will focus on several extensions and technical improvements to enable the system’s utilization in complex real-world applications. These include applying the scalable swarm system to smart and collaborative agriculture, time-sensitive missions such as search-and-rescue or military operations, and swarm operations in space. The following subsections discuss the technical contributions and novelty of the integrated HyRoS system in comparison with other existing systems and also acknowledge the limitations and potential solutions.

### Qualitative comparison to existing systems

We considered several relevant existing works integrating physical and virtual components for swarm or multi-robot systems. The Unity 3D software was used in MR experimentation for autonomous vehicle testing under diverse scenarios with virtual inputs in a previous study^64^. In our case, the HyRoS system utilized MR to test virtual targets and model swarm behavior. Both cases leverage MR to enhance safety by reducing the number of moving vehicles involved. However, there are qualitative differences; the work in^64^ does not utilize VR/MR visualization techniques, relying solely on standard 2D displays. In contrast, using HoloLens in our HyRoS system provides an immersive experience, visualizing the operation of physical and virtual vehicles in the real-world testing location. Similar studies have employed the Autonomous Intersection Management (AIM) simulator to provide simulated intersection data to real vehicles in a physical intersection^65^. These works focus on a single physical vehicle with simulated inputs, while we focus on a hybrid swarm composed of both physical and virtual robots.

Another study implemented a similar application, showcasing an MR hybrid swarm control architecture for Manned-Unmanned Teaming (MUM-T)^28^. This system employed HoloLens for gesture control, like the HyRoS system, while notable differences exist. The MUM-T system utilizes an external multi-camera setup for monitoring the test environment and lacks GPS-based real-world synchronization present in HyRoS. While holographic copies of some agents could be visualized in MUM-T, the virtual agents in our system can execute the perform the algorithms as physical agents using simulated data in the real world. These key differences emphasize the novelty of the HyRoS system. Another relevant study^66^ involved a UAV embedded with fire-detection algorithms in a virtual-physical hybrid system for testing. The UAV received simulated input data of the environment and nearby fires generated in Unity 3D, responding as it would in the real world. The HyRoS system shares a similar function while employing multiple UAVs and UGVs simultaneously to respond to simulated inputs with real-world responses visualized directly alongside the HoloLens view to observe the swarm’s behavior. In another study^67^, MR’s utility is limited to visualizing waypoints on a virtual surrogate before executing the motion with the physical robot.

The swarm metaverse implementation tackles swarm control and modeling challenges through a system that combines physical and simulated environments^68^. Digital twins of real robots in the simulated environment allow for evaluating planned motions for risk avoidance before execution. Both physical swarm members and their digital twins exchange position updates and information. Gesture control methods, observed by an external camera with visualization on a desktop computer, are used for both low and high levels of control. In comparison to the HyRoS system, both utilize gesture commands for swarm control with varying autonomy levels, but the implementation and methodologies differ, such as the unique shepherding method in the swarm metaverse case. The HyRoS system’s key strengths lie in its synchronization between physical and virtual worlds and real-space visualization using MR head-mounted displays, which were not within the scope of the work inswarm metaverse implementation^68^. Additionally, while both cases involve virtual and physical swarm members, their purposes diverge; the swarm metaverse uses virtual agents as digital twins of real-world robots, whereas the HyRoS system allows virtual agents to function independently in the simulated world as representative swarm members that respond to simulated stimuli and targets.

### Limitations and future work

In the current work, the demonstrations primarily involved virtual agents, with only two physical robots being utilized. However, to comprehensively assess the flexibility and configurability of the system, it is crucial to incorporate a larger number of physical robots with varying types and capabilities. This expansion would facilitate more extensive experiments and provide a deeper understanding of the system’s real-world performance. To overcome this limitation and propel the research forward, ongoing efforts are focused on equipping additional physical robots. These robots will allow for a more comprehensive exploration of the system’s capabilities and potential applications. This endeavor brings us closer to achieving our long-term research goal of establishing a robust and practical real-world swarm system.

Currently, the robotic agents within the swarm are equipped with a predefined set of embedded swarm algorithms that govern their collective behaviors. These algorithms can be easily replaced or updated to improve the overall capabilities of the swarm. However, further research is required to effectively support more complex swarm operation scenarios. The system involves a human planner defining and transmitting a task list to the swarm, which autonomously allocates the tasks to subgroups within the swarm for execution. However, a limitation arises when assuming that all tasks within the list are executable by the swarm. The planner may possess limited knowledge and access to real-time information about the environment, which can lead to the assignment of non-executable tasks. Additionally, the desired robots for operation may not be directly accessible but connected through a local swarm network. To tackle these challenges, it becomes important to evaluate the system’s reliability in such scenarios. This involves assessing the system’s ability to handle non-executable tasks and ensuring the connectivity and accessibility of specific robots through the swarm network.

Furthermore, the demonstrations in the current work primarily focused on simple task lists where each task only required one type of robot (UGVs or UAVs). However, in more complex scenarios, tasks may necessitate collaboration among different types of robots. To support such scenarios, it is necessary to update the task allocation algorithm, enabling agents to recruit target agents with specific capabilities. This extension would allow the formation of task groups comprising both UGVs and UAVs (or different types of UGVs/UAVs) based on the required number of each type of robot specified in the task description. We note that forming a task group with heterogeneous agents might take significantly longer, particularly in cases where the swarm forms a loosely connected network. To address this challenge, further experiments should be conducted to evaluate the task allocation function under such conditions and assess its performance in forming task groups with diverse types of robots. These developments would significantly enhance the system’s capabilities and enable it to handle more complex tasks that require collaboration among different types of robots within the system.

### Supplementary Information

Supplementary Information.

## Data Availability

All data generated or analyzed during this study are included in this published article. All data and materials as well as software application or custom code support their published claims and comply with field standards.
